# Fibrotic Patterns and Diagnostic Correlates in Hypersensitivity Pneumonitis: Clinical, Radiologic, and Hematologic Insights

**DOI:** 10.3390/diagnostics15243137

**Published:** 2025-12-09

**Authors:** Esma Sevil Akkurt, Berna Akıncı Ozyurek, Kerem Ensarioglu, Tugce Sahin Ozdemirel, Ozlem Duvenci Birben, Hakan Erturk, Tunahan Dolmus

**Affiliations:** 1 Department of Pulmonology, University of Health Sciences, Ankara Dr. Abdurrahman Yurtaslan Oncology Training and Research Hospital, Ankara 06300, Türkiye; drozlemd.birben@gmail.com; 2 Department of Pulmonology, University of Health Sciences, Ankara Atatürk Sanatorium Training and Research Hospital, Ankara 06300, Türkiye; drberna_1982@yahoo.com (B.A.O.); kerem.ensarioglu@gmail.com (K.E.); drtugcesahin@gmail.com (T.S.O.); tunam1970@gmail.com (T.D.); 3 Department of Radiology, University of Health Sciences, Ankara Atatürk Sanatorium Training and Research Hospital, Ankara 06300, Türkiye; ert.hakan@gmail.com

**Keywords:** basophilia, bird products, fibrotic disease, hypersensitivity pneumonitis

## Abstract

**Background:** Hypersensitivity pneumonitis (HP) is an interstitial lung disease characterized by immune-mediated inflammation and variable degrees of fibrosis. **Aims:** To evaluate the clinical, radiological, and hematological features of patients diagnosed with HP. Study Design: Retrospective cross-sectional study. **Methods:** We included 100 patients diagnosed and followed for HP between 2020 and 2024. Demographic characteristics, pulmonary function test results, diffusing capacity, six-minute walk test findings, antigen exposure history, and high-resolution computed tomography (HRCT) patterns were retrospectively analyzed. **Results:** The mean age was 63 ± 14 years, with equal sex distribution. Sixty-five percent of patients had identifiable antigen exposure, predominantly related to birds or bird products (86.4%). Surgical lung biopsy confirmed the diagnosis in 29% of cases. The most common HRCT findings were reticulation (87%), ground-glass opacities (84.7%), and centrilobular nodules (75%); fibrotic features were present in 48% of patients. Glucocorticoids were the main treatment (77%), and antifibrotic therapy was used in 20% of cases. Neutrophil count showed a modest positive correlation with honeycombing (r = 0.27, *p* = 0.025). Basophil count demonstrated a mild association with bird-related antigen exposure (r = 0.26, *p* = 0.035). **Conclusions:** Peripheral neutrophil and basophil counts showed weak but statistically significant associations with fibrotic HRCT features and exposure patterns. These exploratory findings suggest that routinely available hematologic parameters may provide supportive information alongside radiologic and clinical data. Prospective studies are needed to validate their diagnostic and prognostic relevance in HP.

## 1. Introduction

Hypersensitivity pneumonitis (HP) is an inflammatory and fibrotic disease that affects lung parenchyma and small airways. It is usually caused by an immune-mediated reaction triggered by the inhalation of a known or unknown antigen in susceptible individuals. Common causes include avian proteins, microbial agents such as fungi and bacteria, and low-molecular-weight chemicals [1]. Due to the prognostic impact of fibrosis, HP is now classified as fibrotic or nonfibrotic according to the American Thoracic Society, Japanese Respiratory Society, and Asociación Latinoamericana del Tórax (ATS/JRS/ALAT) guidelines [2].

HP is a complex interstitial lung disease caused by repeated inhalation of various environmental antigens. Although the identification of the causative antigen is critical for both diagnosis and management, it remains a major challenge in clinical practice. In many cases, patients are unaware of the source of exposure, or the exposure history is difficult to obtain. Even with detailed questionnaires and imaging, the antigen may remain unidentified in a significant proportion of patients. Failure to identify and eliminate the inciting antigen has been associated with worse prognosis and progression to fibrosis. In patients known to have been exposed to suspected HP triggers, HP should be considered if there is clinical and radiographic evidence of interstitial lung disease and alternative diagnoses can be excluded. The primary goal of evaluation is to identify the triggering agent [3]. Other diagnostic steps include determining the severity of respiratory distress, evaluating characteristic radiographic findings, and bronchoalveolar lavage (BAL) features. When the diagnosis remains uncertain, a multidisciplinary evaluation with a decision to obtain tissue samples to confirm the diagnosis may be considered.

High-resolution computed tomography (HRCT) findings vary depending on whether HP is fibrotic or nonfibrotic. Nonfibrotic HP is typically associated with ground-glass opacities, although HRCT can be normal due to the transient nature of these changes. Air trapping (mosaic attenuation) may be seen on expiratory–inspiratory comparison. Ground-glass areas are usually few and small. BAL is the most sensitive method to detect alveolitis in suspected cases. A BAL lymphocytosis >20% is a supportive, though nonspecific, finding [4]. The main treatment is antigen avoidance and symptomatic support. Additional therapy may be needed in severe cases [5].

Recent studies have emphasized the clinical importance of integrating radiological, physiological, and immunological parameters to better define HP phenotypes [6]. Moreover, inflammatory cell profiles in peripheral blood may provide additional clues about disease behavior. Basophils are rare granulocytes that play a crucial role in allergic inflammation and Th2-mediated immune responses. They release histamine, leukotrienes, and cytokines such as IL-4 and IL-13, which can contribute to airway inflammation and remodeling. Recent data suggest that elevated peripheral blood basophil counts may reflect systemic antigenic stimulation, particularly in hypersensitivity-related lung diseases [7,8]. However, their role in HP remains under-investigated. Alongside neutrophils and lymphocytes, basophils may serve as peripheral biomarkers reflecting the immunologic burden and possibly the nature of antigen exposure.

In addition, recent multicenter radiological evidence has strengthened the diagnostic value of HRCT small-airway findings. A large multicenter study demonstrated that the extent of air trapping was the strongest radiologic predictor for a multidisciplinary diagnosis of HP, and that quantitative assessment of small-airway abnormalities had high diagnostic accuracy in distinguishing HP from other interstitial lung diseases [9]. These findings highlight the importance of airway-centered changes as key diagnostic features, particularly in cases with non-specific parenchymal involvement.

This study aims to evaluate the clinical, radiological, and laboratory characteristics of patients diagnosed with HP in our center, with a particular focus on the relationship between inflammatory markers and radiological fibrosis. By integrating clinical, radiologic, and hematologic data, we sought to contribute to the understanding of HP phenotypes and their prognostic implications, especially in patients where a clear causative antigen cannot be identified.

## 2. Materials and Methods

### 2.1. Study Design and Population

This retrospective cross-sectional study included 100 consecutive patients diagnosed with hypersensitivity pneumonitis at a tertiary interstitial lung disease (ILD) center between January 2020 and January 2024. All patients evaluated in the ILD outpatient clinic during the study period who met the diagnostic criteria for HP were included; therefore, no randomization or additional stratification was applied. The study was approved by the Institutional Ethics Committee (Approval No: 2024-BÇEK/96; approval date: 12 June 2024), and all procedures were conducted in accordance with the Declaration of Helsinki. The requirement for individual informed consent was waived due to the retrospective design of the study and the use of anonymized patient data.

Patients aged ≥18 years with clinical, radiological, and/or histopathological findings consistent with HP were included. Cases with insufficient records, alternative diagnoses, or corticosteroid-related hyperglycemia were excluded. HP diagnoses were established through real-time multidisciplinary discussion (MDD), involving pulmonologists, thoracic radiologists, and pathologists. No retrospective reclassification was performed, and no previously established HP diagnosis was overturned during re-evaluation.

Diagnostic confidence levels (definite, high-confidence, moderate, low-confidence HP) were assigned according to ATS/JRS/ALAT guideline criteria and recorded in the study dataset.

### 2.2. Data Collection and Clinical Parameters

Data were collected using a standardized electronic extraction form applied uniformly across the study period. Demographic variables, comorbidities, smoking history, exposure characteristics, and symptom profiles were retrieved from medical records.

Exposure history was obtained using a structured environmental questionnaire routinely implemented in our ILD clinic, covering occupational, household, avian, mold, and chemical exposures. The same questionnaire format was used throughout the entire study period.

Pulmonary function tests (PFTs), including forced vital capacity (FVC) and diffusing capacity for carbon monoxide (DLCO), and six-minute walk test (6MWT) results were recorded at the time of diagnosis.

### 2.3. Radiological Evaluation

HRCT scans obtained at diagnosis were evaluated by two thoracic radiologists with >10 years of ILD experience, blinded to clinical and laboratory data. Radiologic patterns were categorized as typical, compatible, or indeterminate for HP according to ATS/JRS/ALAT criteria.

Fibrosis was defined by the presence of traction bronchiectasis or honeycombing. A semiquantitative visual grading system was used—mild (<25%), moderate (25–50%), or severe (>50%) involvement. Interobserver discrepancies were resolved through consensus. Diagnostic confidence scores were recorded during the MDD process.

### 2.4. Laboratory Parameters

Peripheral blood tests performed at the initial diagnostic visit and prior to initiation of systemic corticosteroids or immunosuppressive therapy were included. Complete blood count (CBC) parameters—including neutrophil, lymphocyte, eosinophil, and basophil counts—along with C-reactive protein (CRP) and erythrocyte sedimentation rate (ESR) were collected from the hospital laboratory database.

### 2.5. Histopathological Confirmation

During the study period, cryobiopsy was not available in our institution. Conventional transbronchial forceps biopsy (TBB) was not preferred because of its limited diagnostic yield in fibrotic interstitial lung diseases, including fibrotic HP. Therefore, when tissue sampling was required, histopathologic confirmation was obtained exclusively by surgical lung biopsy (VATS wedge biopsy). A total of 29 patients (29%) underwent VATS biopsy. Among patients without identifiable antigen exposure, 14 required surgical biopsy specifically for diagnostic clarification. Histopathologic patterns consistent with HP included cellular interstitial pneumonitis, bronchiolocentric fibrosis, and poorly formed granulomas.

### 2.6. Statistical Analysis

Statistical analyses were conducted using IBM SPSS Statistics 26.0. Normality was assessed using the Shapiro–Wilk test and visual inspection of histograms. Continuous variables were presented as mean ± standard deviation or median (interquartile range); categorical variables as frequencies and percentages.

Group comparisons were performed using Student’s *t*-test or Mann–Whitney U test as appropriate. For exploratory purposes, correlation analyses (Pearson or Spearman) were applied, including correlations involving binary clinical variables, recognizing that these analyses serve supportive rather than confirmatory statistical functions. A *p*-value < 0.05 indicated statistical significance.

## 3. Results

### 3.1. Demographic and Clinical Characteristics

A total of 100 patients were included in the study, with an equal distribution of males and females (50% each). The mean age was 63 ± 14 years (range: 22–85). Regarding smoking status, 49 patients (49%) were current or former smokers, while 51 patients (51%) were lifelong nonsmokers.

Among comorbidities, systemic arterial hypertension was observed in 37% of patients and diabetes mellitus in 23%. Respiratory comorbidities included asthma in 10% and chronic obstructive pulmonary disease (COPD) in 9% of the cohort. Coronary artery disease was present in 16% of patients. Detailed demographic and clinical characteristics are presented in Table 1.

### 3.2. Antigen Exposure and Diagnostic Approach

A total of 65 patients (65%) had a clearly identifiable history of environmental or occupational antigen exposure. Among these, exposure to birds or bird-related products was the most common cause and accounted for 86.4% of all known exposures, followed by mold exposure (9.1%) and chemical vapor exposure (4.6%). All exposure histories were obtained using a structured environmental questionnaire routinely implemented in our ILD clinic.

Thirty-five patients (35%) had no identifiable antigen despite detailed interviews, environmental history, and questionnaire-based assessment. This group required a more extensive diagnostic work-up, and 14 of these 35 patients (40%) underwent surgical lung biopsy to achieve diagnostic confirmation. Typical histopathologic features consistent with hypersensitivity pneumonitis were present in all biopsy-confirmed cases within this subgroup.

Overall, 29 patients (29%) in the entire cohort underwent surgical lung biopsy, while the remaining 71 patients (71%) received a clinical–radiologic diagnosis based on compatible HRCT findings with or without supportive BAL results. Exposure characteristics and diagnostic distribution are summarized in Table 2.

### 3.3. Radiological Findings

The most frequently observed HRCT abnormalities were reticulation (87%), ground-glass opacities (84.7%), and centrilobular nodules (75%). Mosaic attenuation—reflecting small-airway involvement and air trapping—was present in 56 patients (56%) and represented one of the characteristic radiologic features of the cohort. Fibrotic findings, specifically traction bronchiectasis and honeycombing, were identified in 48 patients (48%), corresponding to the subgroup classified as fibrotic HP.

Radiologic classification according to ATS/JRS/ALAT criteria showed a typical HP pattern in 16 patients (16%), a compatible/suggestive pattern in 73 patients (73%), and an atypical pattern in 11 patients (11%). Importantly, these 11 atypical cases were not diagnosed based on HRCT appearance alone. Six patients (54.5%) had a clearly identified environmental or occupational antigen exposure, and four patients (36.4%) underwent surgical lung biopsy that supported the diagnosis. The remaining patient (9.1%) received a final diagnosis of HP through multidisciplinary discussion (MDD), integrating exposure history, clinical–radiological concordance, and supportive BAL findings. Thus, all atypical cases were diagnosed through a comprehensive, guideline-based diagnostic approach rather than radiology in isolation.

Fibrosis severity was assessed using a semiquantitative visual grading system and was available for all 48 patients with fibrotic HP. Based on this grading, fibrosis was classified as mild in 19 patients (39.6%), moderate in 17 (35.4%), and severe in 12 (25.0%). Interobserver agreement between the two thoracic radiologists for fibrosis grading was excellent (κ = 0.82), confirming the reproducibility of the radiologic assessment. A detailed distribution of HRCT findings and radiologic patterns is presented in Table 3.

A total of 48 patients (48%) had fibrotic HP and 52 (52%) had non-fibrotic disease. In the subgroup analysis, patients with fibrotic HP demonstrated greater physiological impairment; however, most differences did not reach statistical significance. Six-minute walk distance was lower in the fibrotic group (383 ± 124 m vs. 428 ± 95.8 m in non-fibrotic HP, *p* = 0.127). FVC values were modestly reduced in the fibrotic group (76.2% vs. 81.0%, *p* = 0.275), and DLCO was slightly lower (median 62.5% vs. 64%, *p* = 0.198), while DLCO/VA values were similar between the two groups (*p* = 0.924). BAL lymphocyte percentages did not differ significantly (23.8% vs. 22.5%, *p* = 0.827). These functional and BAL findings are summarized in Table 4.

In contrast, hematologic parameters showed more prominent differences between the groups. Patients with fibrotic HP had significantly higher WBC counts (9.53 ± 3.28 vs. 8.23 ± 2.32 × 10^3^/µL, *p* = 0.024) and neutrophil counts (6.14 ± 2.79 vs. 4.87 ± 2.11 × 10^3^/µL, *p* = 0.011), indicating increased systemic inflammatory burden. Hemoglobin levels were also modestly but significantly higher in fibrotic patients (14.54 ± 1.63 vs. 13.77 ± 1.79 g/dL, *p* = 0.024), possibly reflecting chronic hypoxemia-related compensatory mechanisms. Other hematologic indices—including lymphocyte count (*p* = 0.810), NLR (*p* = 0.132), PLR (*p* = 0.690), basophil count (*p* = 0.344), eosinophil count (*p* = 0.487), platelet count (*p* = 0.558), and RDW (*p* = 0.334)—did not differ significantly between groups. The complete functional, BAL, and hematologic comparison is presented in Table 4.

### 3.4. Treatment and Outcomes

Glucocorticoids were administered to 77 patients (77%), predominantly among those with non-fibrotic or inflammatory radiologic features. Antifibrotic therapy (pirfenidone or nintedanib) was initiated in 20 patients (20%), all of whom demonstrated fibrotic HRCT findings at baseline. Combination therapy with both glucocorticoids and antifibrotics was used in 12 patients (12%), representing cases with progressive fibrotic disease despite initial treatment.

Eight patients (8%) were managed with antigen avoidance and clinical follow-up alone due to mild disease without physiologic impairment.

Disease progression within one year occurred in 21 patients (21%). Progression was defined as any of the following: a ≥10% absolute decline in FVC, a ≥15% decline in DLCO, or radiologic progression characterized by increased fibrosis on HRCT.

The one-year mortality rate was 9%, consistent with prior ILD cohorts. Most deaths were attributed to respiratory failure or infectious complications. Treatment distributions and clinical outcomes are summarized in Table 5.

### 3.5. Correlation Analyses

Exploratory correlation analyses were performed to assess potential associations between peripheral blood indices and radiologic or functional variables. A positive correlation was observed between neutrophil count and the presence of honeycombing (r = 0.27, *p* = 0.025), indicating that higher neutrophil levels were more frequently seen in patients with fibrotic HRCT features. Basophil count showed a mild positive correlation with exposure to birds and bird-related products (r = 0.26, *p* = 0.035).

No significant correlations were identified between eosinophil count and radiologic or pulmonary function parameters. Similarly, the negative correlation between neutrophil count and mosaic attenuation did not reach statistical significance (r = −0.24, *p* = 0.054) and was interpreted with caution.

Patients receiving antifibrotic therapy demonstrated slightly higher baseline basophil levels compared with those on glucocorticoid therapy alone, although this observation was exploratory and not designed to determine causality.

All correlation findings are summarized in Table 6.

## 4. Discussion

In this retrospective study, we evaluated 100 patients with hypersensitivity pneumonitis to examine the relationship between clinical features, radiologic patterns, and peripheral inflammatory markers. Fibrotic changes on HRCT were common, and certain hematologic indices—particularly neutrophil and basophil counts—showed statistically significant but modest associations with specific radiologic and exposure-related variables. These findings should be interpreted as exploratory, but they indicate that simple hematologic markers may contribute additional context to the clinical evaluation of HP.

Although recent ATS and CHEST guidelines recommend CRYO-TBB as a less invasive diagnostic approach with high diagnostic yield, cryobiopsy was not available in our institution during the study period. In addition, conventional transbronchial forceps biopsy has limited diagnostic yield in fibrotic ILDs and was therefore not routinely used. For this reason, VATS wedge biopsy was performed in selected patients when histopathologic confirmation was needed, reflecting institutional limitations rather than preference for SLB as a first-line modality.

HP is a heterogeneous interstitial lung disease with considerable variability in causative antigens and disease behavior [10,11]. Consistent with previous studies, bird-related exposure was the predominant trigger in our cohort [12]. The strong representation of avian antigens may partly explain the mild correlation observed between basophil count and exposure, as chronic antigenic stimulation could influence granulocyte patterns. Nonetheless, this association should be viewed cautiously given its modest effect size.

The demographic characteristics of our cohort were comparable to those reported in recent large series [13,14,15,16,17]. Pulmonary function tests demonstrated predominantly restrictive physiology and reduced DLCO, in line with existing data [18]. Although short-term spirometry remained stable in most patients, approximately one-third experienced an early decline in DLCO or FVC, consistent with evidence that reductions in diffusing capacity or forced vital capacity predict worse outcomes in chronic HP [19,20,21,22].

Radiologically, the most frequent findings included reticulation, ground-glass opacities, centrilobular nodules, and mosaic attenuation. These features reflect bronchiolocentric inflammation and small airway involvement, which are characteristic of HP [6]. Recent multicenter data emphasize that air trapping and small-airway quantitative metrics are strong predictors for multidisciplinary diagnosis of HP [10]. The high prevalence of mosaic attenuation in our cohort supports the central role of small airway disease in the phenotypic expression of HP.

Fibrotic features were present in 48% of patients, suggesting that many individuals presented with chronic or advanced disease. Fibrosis severity was associated with reduced DLCO and functional impairment, which is consistent with established concepts regarding gas exchange limitations in fibrotic ILDs. Neutrophil count demonstrated a statistically significant but weak correlation with honeycombing. Although this association is biologically plausible, the modest effect size indicates that it should be interpreted as supportive rather than mechanistically definitive.

In the subgroup comparison of fibrotic and non-fibrotic HP, the most distinct differences emerged in hematologic markers rather than in functional or BAL parameters. Patients with fibrotic HP demonstrated significantly higher WBC and neutrophil counts, indicating a more pronounced systemic inflammatory response in the fibrotic phenotype. The significantly higher hemoglobin values in the fibrotic group may also reflect chronic hypoxemia-related compensatory mechanisms commonly observed in progressive fibrotic lung disease. In contrast, functional indices such as FVC, DLCO, DLCO/VA, and six-minute walk distance, as well as BAL lymphocyte percentages, did not differ significantly between the groups. These findings suggest that functional tests and BAL profiles alone may have limited ability to distinguish between fibrotic and non-fibrotic HP in chronic disease stages.

BAL lymphocytosis is commonly used to support the diagnosis of HP; however, its diagnostic specificity remains limited. In our cohort, BAL lymphocyte percentages did not differ significantly between fibrotic and non-fibrotic cases. Similar discrepancies have been reported previously [2,23]. Differential BAL granulocyte data were not available, preventing direct comparison between airway and blood cell patterns. Nevertheless, the combination of clinical, radiologic, and laboratory information provided sufficient diagnostic clarity in most cases.

Treatment strategies in our cohort reflected current practice patterns. Corticosteroids were used in the majority of patients, while antifibrotic therapy was applied in those with progressive fibrotic disease. Evidence supporting pharmacologic therapy in HP remains limited, though data from the INBUILD and other studies support the use of antifibrotics in progressive fibrosing ILDs [24,25,26,27,28]. In our cohort, patients receiving antifibrotics tended to have more advanced fibrosis. However, all treatment-related observations remain descriptive and should not be interpreted as causal due to potential confounding factors.

This study has several limitations. First, the retrospective and single-center design limits generalizability. Antigen identification relied primarily on clinical history and structured questionnaires, as serologic and provocation tests were not consistently available. Although certain correlations reached statistical significance, effect sizes were modest, reflecting the biological heterogeneity of HP. Residual confounding from disease chronicity, exposure variability, and treatment differences cannot be excluded. BAL differential data were incomplete, and prior COVID-19 infection status could not be assessed despite its potential relevance to fibrotic outcomes. Finally, although a semiquantitative fibrosis grading system was applied, a formal Goh score could not be used due to variability in imaging protocols. Another limitation of our study is that 11 patients had atypical HRCT findings without histopathological confirmation; however, all were diagnosed as HP based on compatible exposure history, clinical–radiological concordance, supportive BAL findings when applicable, and a multidisciplinary discussion consensus. In addition, the statistical power of the subgroup analyses comparing fibrotic and non-fibrotic HP was limited due to the sample size, and therefore these results should be interpreted with caution.

Future prospective studies incorporating standardized exposure tools, quantitative HRCT metrics, and longitudinal hematologic analysis will help clarify the prognostic significance of peripheral inflammatory markers in HP. Even modest associations may help generate hypotheses for mechanistic and translational research aimed at better defining HP phenotypes.

## 5. Conclusions

This study evaluated the clinical, radiological, and hematological characteristics of patients with hypersensitivity pneumonitis, underscoring the central role of thorough exposure assessment in diagnosis. Neutrophilia showed a modest association with fibrotic HRCT features, while basophilia was weakly correlated with bird-related antigen exposure. Although these findings are exploratory, they suggest that simple, routinely available laboratory parameters may provide supportive information when interpreting radiologic and clinical features of HP. Future prospective studies are needed to validate these associations, clarify their prognostic value, and define their relevance to disease monitoring and phenotyping.

## Figures and Tables

**Table 1:** Demographic and Clinical Characteristics.

Parameters		*N* (%)
Gender	Female	50
	Male	50
Age (years, ±SD)		63 (±14)
Smoking	Nonsmoker	51 (51%)
	Current Smoker	49 (49%)
Chronic Obstructive Pulmonary Disease	None	91 (91%)
	Present	9 (9%)
Asthma	None	90 (90%)
	Present	10 (10%)
Coronary Arterial Disease	None	84 (84%)
	Present	16 (16%)
Diabetes Mellitus (excluding treatment-related)	None	77 (77%)
	Present	23 (23%)
Systemic Arterial Hypertension	None	63 (63%)
	Present	37 (37%)

SD: Standard Deviation, Diabetes Mellitus definition included both type 1 and type 2 diabetes, yet excluded treatment-related ones.

**Table 2:** Distribution of Known and Unknown Antigen Exposure in Patients with HP.

Parameter	*n* (%)	Description/Notes
Known antigen exposure	65 (65%)	Patients with a clearly identified environmental or occupational exposure
Unknown antigen exposure	35 (35%)	No identifiable antigen despite detailed history and evaluation
Type of antigen exposure		
• Bird or bird-product exposure	56 (86.4%)	Pigeons, parrots, and domestic birds most frequently implicated
• Mold exposure	6 (9.6%)	Mold contamination in household or workplace environments
• Chemical exposure	3 (4%)	Low-molecular-weight compounds (e.g., isocyanates)
**Diagnostic confirmation**		
• Surgical lung biopsy performed	29 (29%)	Histopathologic confirmation of HP pattern
• Clinical–radiologic diagnosis (HRCT ± BAL)	71 (71%)	Diagnosis based on HRCT findings and exposure history

Data are expressed as number (percentage). Percentages refer to the total number of patients with known exposure. Acronyms used: HP, hypersensitivity pneumonitis. HRCT: High-Resolution Computed Tomography. BAL: Bronchoalveolar Lavage.

**Table 3:** High-Resolution Computed Tomography Findings of the Study Population.

HRCT Finding	*n* (%)
Reticulation	87 (87%)
Ground-glass opacities	84 (84.7%)
Centrilobular nodules	75 (75%)
Mosaic attenuation (air trapping)	56 (56%)
Fibrosis (traction bronchiectasis/honeycombing)	48 (48%)
**HRCT Pattern**	
• Typical HP pattern	16 (16%)
• Compatible HP pattern	73 (73%)
• Atypical HP pattern	11 (11%)

**Table 4:** Subgroup Comparison of Fibrotic and Non-Fibrotic HP.

Parameter	Non-Fibrotic HP (*n* = 52)	Fibrotic HP (*n* = 48)	*p*-Value
BAL lymphocyte (%)	22.5 ± 17.4	23.8 ± 22.6	0.827
FEV1 (%)	79.6 ± 20.8	79.3 ± 24.5	0.951
FVC (%)	81.0 ± 19.0	76.2 ± 21.8	0.275
DLCO (%)	64	62.5	0.198
DLCO/VA	94.1 ± 20.7	93.6 ± 20.9	0.924
6 min walk (m)	428 ± 95.8	383 ± 124.0	0.127
WBC (×10^3^/µL)	8.23 ± 2.32	9.53 ± 3.28	0.024 *
Neutrophil (×10^3^/µL)	4.87 ± 2.11	6.14 ± 2.79	0.011 *
Lymphocyte (×10^3^/µL)	2.44 ± 0.79	2.40 ± 0.95	0.810
NLR	2.38 ± 2.55	3.12 ± 2.35	0.132
PLR	121.90 ± 60.86	127.06 ± 67.87	0.690
Basophil (×10^3^/µL)	0.04 ± 0.02	0.05 ± 0.02	0.344
Eosinophil (×10^3^/µL)	0.44 ± 1.71	0.28 ± 0.22	0.487
HGB (g/dL)	13.77 ± 1.79	14.54 ± 1.63	0.024 *
Platelets (×10^3^/µL)	266.93 ± 87.59	258.04 ± 63.19	0.558
RDW (%)	14.47 ± 1.78	14.89 ± 2.50	0.334

Statistically significant differences (* *p* < 0.05) are shown in bold.

**Table 5:** Treatment Characteristics and Clinical Outcomes.

Parameter	*n* (%)	Description/Notes
Glucocorticoid therapy	77 (77%)	Systemic corticosteroids were the main treatment modality
Antifibrotic therapy	20 (20%)	Pirfenidone or nintedanib used in progressive fibrotic cases
Combination therapy	12 (12%)	Patients receiving both glucocorticoid and antifibrotic treatment
Observation only	8 (8%)	Mild cases managed with antigen avoidance and clinical monitoring
Disease progression (1-year)	21 (21%)	Defined as decline in PFTs or worsening on HRCT
Mortality (1-year)	9 (9%)	Deaths attributed to respiratory failure or infection

Disease progression and mortality rates were evaluated within one year following diagnosis. Acronyms used: PFT = pulmonary function test; HRCT = high-resolution computed tomography.

**Table 6:** Correlation Between Hematologic Parameters and Radiological/Functional Findings.

Parameter	Correlated Variable	Correlation Coefficient (r)	*p*-Value
Neutrophil count	Honeycombing (fibrosis on HRCT)	0.27	**0.025**
Neutrophil count	Mosaic attenuation (air trapping)	−0.24	0.054
Basophil count	Exposure to birds and bird products	0.26	**0.035**
Eosinophil count	Radiologic fibrosis extent	0.09	0.412
CRP level	DL_CO_ (%)	−0.18	0.082

Abbreviations: HRCT, high-resolution computed tomography; CRP, C-reactive protein; DL_CO_, diffusing capacity for carbon monoxide. Note: Pearson or Spearman correlation coefficients were used according to data distribution. Statistically significant correlations (*p* < 0.05) are shown in bold.

## Data Availability

The data presented in this study are available from the corresponding author upon reasonable request. The data are not publicly available due to privacy and ethical restrictions.
